# A nocturnal atmospheric loss of CH_2_I_2_ in the remote marine boundary layer

**DOI:** 10.1007/s10874-015-9320-6

**Published:** 2015-10-05

**Authors:** Lucy J. Carpenter, Stephen J. Andrews, Richard T. Lidster, Alfonso Saiz-Lopez, Miguel Fernandez-Sanchez, William J. Bloss, Bin Ouyang, Roderic L. Jones

**Affiliations:** 1 Wolfson Atmospheric Chemistry Laboratories, Department of Chemistry, University of York, York, YO10 5DD UK; 2 Atmospheric Chemistry and Climate Group, Institute of Physical Chemistry Rocasolano, Spanish National Research Council (CSIC), 28006 Madrid, Spain; 3 School of Geography, Earth and Environmental Sciences, University of Birmingham, Birmingham, B15 2TT UK; 4 Department of Chemistry, University of Cambridge, Cambridge, CB2, 1EW UK

**Keywords:** di-iodomethane, NO3 radical, Atmosphere, Ocean, Iodine

## Abstract

Ocean emissions of inorganic and organic iodine compounds drive the biogeochemical cycle of iodine and produce reactive ozone-destroying iodine radicals that influence the oxidizing capacity of the atmosphere. Di-iodomethane (CH_2_I_2_) and chloro-iodomethane (CH_2_ICl) are the two most important organic iodine precursors in the marine boundary layer. Ship-borne measurements made during the TORERO (Tropical Ocean tRoposphere Exchange of Reactive halogens and Oxygenated VOC) field campaign in the east tropical Pacific Ocean in January/February 2012 revealed strong diurnal cycles of CH_2_I_2_ and CH_2_ICl in air and of CH_2_I_2_ in seawater. Both compounds are known to undergo rapid photolysis during the day, but models assume no night-time atmospheric losses. Surprisingly, the diurnal cycle of CH_2_I_2_ was lower in amplitude than that of CH_2_ICl, despite its faster photolysis rate. We speculate that night-time loss of CH_2_I_2_ occurs due to reaction with NO_3_ radicals. Indirect results from a laboratory study under ambient atmospheric boundary layer conditions indicate a *k*_CH2I2+NO3_ of ≤4 × 10^−13^ cm^3^ molecule^−1^ s^−1^; a previous kinetic study carried out at ≤100 Torr found *k*_CH2I2+NO3_ of 4 × 10^−13^ cm^3^ molecule^−1^ s^−1^. Using the 1-dimensional atmospheric THAMO model driven by sea-air fluxes calculated from the seawater and air measurements (averaging 1.8 +/− 0.8 nmol m^−2^ d^−1^ for CH_2_I_2_ and 3.7 +/− 0.8 nmol m^−2^ d^−1^ for CH_2_ICl), we show that the model overestimates night-time CH_2_I_2_ by >60 % but reaches good agreement with the measurements when the CH_2_I_2_ + NO_3_ reaction is included at 2–4 × 10^−13^ cm^3^ molecule^−1^ s^−1^. We conclude that the reaction has a significant effect on CH_2_I_2_ and helps reconcile observed and modeled concentrations. We recommend further direct measurements of this reaction under atmospheric conditions, including of product branching ratios.

## Introduction

The potential for tropospheric ozone destruction by iodine was recognized many decades ago (Chameides and Davis 1980) and confirmed later by measurements of the iodine oxide radical (IO) (Alicke et al. 1999; Read et al. 2008; Mahajan et al. 2010a, b; Gómez Martín et al. 2013; Großmann et al. 2013; Dix et al. 2013; Prados-Roman et al. 2015). A ubiquitous global marine boundary layer (MBL) background of around 0.5–1 pptv IO has been established by these measurements. The presence of IO establishes that active atmospheric iodine chemistry is occurring, driven by photochemical breakdown of iodine precursors. Iodine is a significant ozone-depleting agent in the MBL, being responsible for 20–30 % of the total ozone loss in that region (Saiz-Lopez et al. 2014; Prados-Roman et al. 2015; Sherwen et al. 2015). Calculations from global chemistry transport models indicate that globally iodine provides an odd oxygen (Ox) loss of 12–16 % of the total, at around ~720 Tg yr.^−1^ (Saiz-Lopez et al. 2014; Sherwen et al. 2015).

Oceanic emissions of the inorganic gases I_2_ and HOI, produced from ozonolysis of surface iodide (Carpenter et al. 2013; MacDonald et al. 2014), are believed to contribute ~2–2.5 Tg I yr.^−1^, around 80 % of global iodine emissions (Sherwen et al. 2015; Prados-Roman et al. 2015). However, in certain regions - including at high latitudes where surface ocean iodide levels are low, and in locations where wind speeds are high and surface ozone (O_3_(g)) mixing ratios are relatively low - organic gases may contribute 50 % or more of the iodine source (Prados-Roman et al. 2015). There are few oceanic flux measurements of CH_2_I_2_ and CH_2_ICl, but available data suggest that they contribute ~0.11 and 0.17 Tg I yr.^−1^ to the global atmosphere (Jones et al. 2010) similar in total to methyl iodide (CH_3_I) emissions (Carpenter et al. 2014).

Both CH_2_I_2_ and CH_2_ICl are known to have short photolysis lifetimes in the atmosphere of ~5 min and ~1 h at midday in mid-latitudes, respectively (Rattigan et al. 1997; Roehl et al. 1997; Mössinger et al. 1998), leading to strong diurnal cycles (Carpenter et al. 1999; Jones et al. 2010). Photolysis is assumed to be the sole driver of atmospheric loss of these compounds, and in the presence of chloride ions likely dominates aqueous loss also (Jones and Carpenter 2005; Martino et al. 2005). Laboratory determination of the photolysis rates in CH_2_I_2_ and CH_2_ICl in seawater indicate aqueous lifetimes at midday in mid- latitudes of 9–12 min for CH_2_I_2_ and 9–13 h for CH_2_ICl (Jones and Carpenter 2005; Martino et al. 2005).

So far, the possibility of night-time losses of the di-halomethanes remains unexplored in terms of analysis of field data and atmospheric modeling. Nakano et al. (2006) performed a kinetic study of the atmospheric reaction of NO_3_ with CH_2_I_2_ (reaction 1) and determined a rate constant of (4.0 ± 1.2) × 10^−13^ cm^3^ molecule^−1^ s^−1^. This would result in a nocturnal lifetime of ~3 h for CH_2_I_2_ in the clean MBL.

1 $$ {\mathrm{NO}}_3+{CH}_2{\mathrm{I}}_2\to \mathrm{Products} $$

However this study was carried out at low pressure (25–100 Torr of N_2_ diluent) and there appear to be no corroborating studies in the literature.

Here we report seawater and air measurements and calculated ocean emissions of CH_2_I_2_ and CH_2_ICl from the eastern tropical Pacific Ocean. We focus on a 4-day period of near constant sea surface temperatures, chlorophyll *a* levels, and wind speeds, whilst sampling water from the North Equatorial Current. Measurements are compared to the 1D Tropospheric Halogen Chemistry Model (THAMO) (Saiz-Lopez et al. 2008) to test understanding of the atmospheric losses of CH_2_I_2_ and CH_2_ICl. We also infer a range of possible values for *k*_CH2I2+NO3_ from kinetic results obtained during a laboratory study of the reaction of the Criegee intermediate CH_2_OO with NO_2_¸which are the first data (to our knowledge) for this reaction obtained under ambient atmospheric boundary layer conditions (Ouyang et al. 2013).

## Methods

### Cruise track and shipboard measurements

**Fig. 1:**
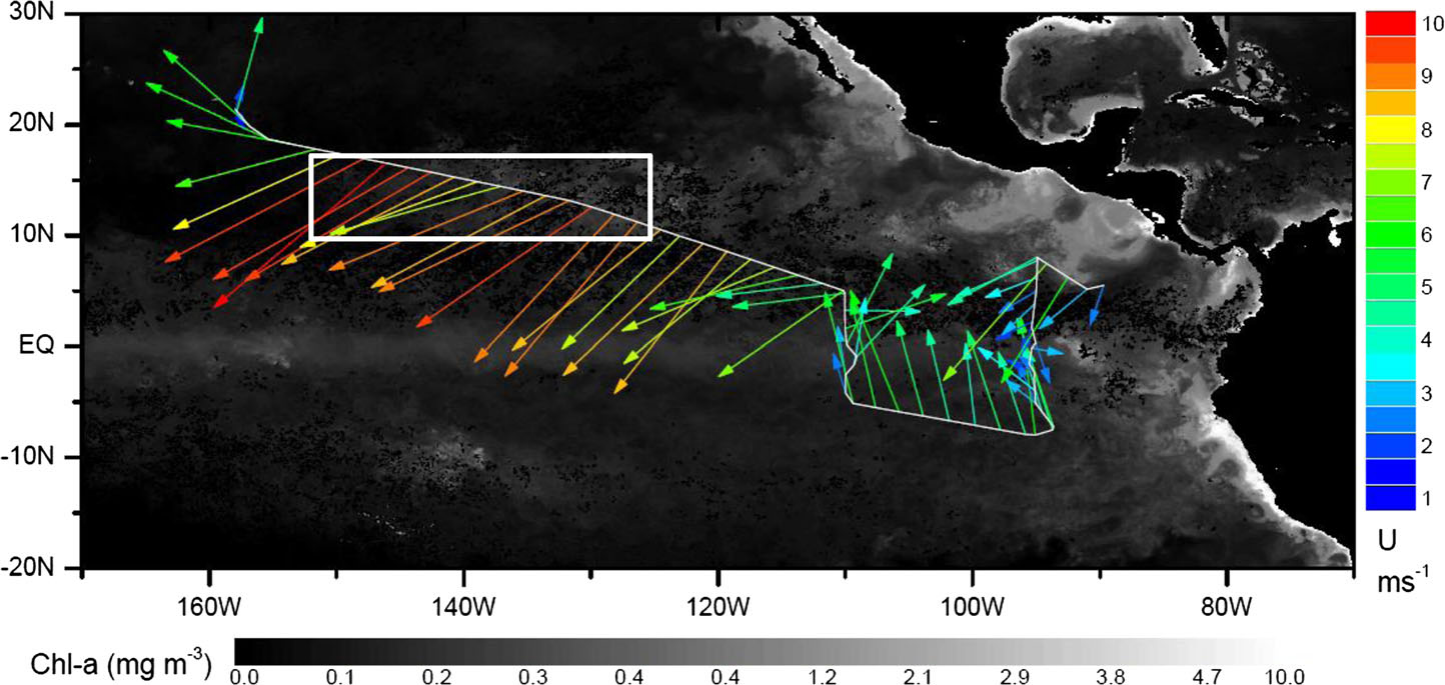
Cruise track of the R/V Ka’imimoana during the TORERO cruise during Jan./Feb. 2012, superimposed on chlorophyll-a background (MODIS) and showing wind speed and direction. The white rectangle shows the part of the cruise track chosen for the case study

The NOAA research vessel *Ka*’*imimoana* sailed from Pearl Harbour, Hawaii to Puntarenas, Costa Rica, via the 95^o^W and 110^o^W Tropical Atmosphere Ocean (TAO) buoy lines from 27th Jan. until 28th Feb. 2012 (Fig. 1). Very short-lived halocarbons (VSLH) were quantified in air and seawater at regular intervals during the cruise.

#### Halocarbon measurements

Air was sampled from the top of the 10 m bow tower through a 60 m sampling line (1/2″ PFA, Swagelok). A diaphragm pump (KNF, NO35.1.2AN.18) was used to pull ~30 L min^−1^ down the line. At 1 m before the diaphragm pump, a T-union connected approximately 50 cm of 1/4″ heated instrument grade stainless steel line (Restek) to an inverted metal bellows pump (Senior Aerospace MB-158) which provided 10 psi of positive pressure to the instrument via ~1.5 m of 1/8″ heated instrument grade stainless steel tubing with a pressure relief valve maintaining flow and draining any water whilst the instrument was not sampling. An ozone analyser (Thermo Scientific Model 49i) drawing 3 L min^−1^ was also connected downstream of the metal bellows pump. Water was removed from the sample stream using a Nafion drier (Permapure, MD-050-72S-2) with a nitrogen counter current.

The absence of any line losses of VSLH using 100 m of 1/2″ PFA sampling line was verified in laboratory tests. A 35 L electropolished cylinder (Essex Cyrogenics) was filled with ambient air at 60 bar using a clean, high-pressure compressor (RIX SA-6). The cylinder air was (1) introduced directly into the instrument, using a pressure regulator and (2) used to over-flow the inlet of the pumped sampling system using 100 m of PFA line. This was performed at an open T-union so as not to influence the pressure in the sample line. The results of multiple analyses of air introduced directly and through the pumped sample line were compared and showed no significant losses (deviations less than the instrument reproducibility) for all of the halocarbons measured.

Calibrations were run daily using 1 L of a National Oceanic and Atmospheric Administration (NOAA) VSLH standard (SX-3570). All analytes in this standard were tested to be stable over 4 years by comparison with subsequently prepared standards, SX-3576 and SX-3581 at 2 and 4 years, respectively. In order to correct for short-term sensitivity changes between calibrations, atmospheric CCl_4_ was used as an internal standard. The air samples (1 L at 100 mL min^−1^), calibration and zero (nitrogen, n6.0) gas streams were selected (Markes, CIA8) and pre-concentrated on a cooled absorbent trap at −30 °C (Markes, Unity2). Samples were desorbed at 300 °C onto a gas chromatograph (GC, Agilent 7890) and separated on a capillary column (Restek, RTX502.2, 30 m, 0.25 mm ID, 1.25 μm) using an oven temperature ramp (40–250 °C, 3 min hold, 20 °C min^−1^ ramp). Eluents were ionized using electron ionization and analysed by an Agilent 5975C mass spectrometer (MS). CH_2_ICl and CH_2_I_2_ were measured using *m*/*z* 176/178 and 268/254 as quantification/qualification ions, respectively.

Surface seawater was sampled from the ship’s seawater inlet, located approx. 2–4 m (depending on sea-state) below the waterline, every 55 min (as well as at various depths from conductivity, temperature, depth (CTD) casts at specific points during the voyage, which are not discussed here). The seawater was pumped through the bow of the ship and through a vortex de-bubbler (Seabird) to remove any air bubbles, at a flow of around 10 L min^−1^ through ½” PFA tubing. A peristaltic pump was used to divert a portion of the water to an automated purge and trap (AutoP&T) system (Andrews et al. 2015). Briefly, the AutoP&T comprises a heated glass purge vessel automatically filled and emptied using a series of valves. A fixed sample volume (20 mL) was filtered (0.45 μm, PTFE filter) and de-gassed at 50 °C using 1 L of zero grade nitrogen at 50 mL min^−1^. Analysis was carried out using a second GC-MS with the same set-up as the air instrument. Similarly to the air measurements, atmospheric CCl_4_ was used to correct for short-term sensitivity changes between calibrations, by rapid sampling of 500 mL of air between each water sample.

#### Ozone measurements

Atmospheric ozone concentrations were measured throughout the cruise using a Thermo Scientific Model 49i Ozone Analyzer. The ozone analyzer was connected to the main air sampling line just downstream of the tee connecting the metal bellows pump.

### Sea-air flux determination

Fluxes of VSLH across the ocean-atmosphere interface were calculated from the seawater-air concentration gradients using the two-layer model (Liss and Slater 1974);

2 $$ F={k}_T\left({C}_w-{C}_g/H\right) $$

and

3 $$ \mathrm{l}/{k}_T=1/{k}_w+1/H{k}_a $$

where *C*_*w*_ and *C*_*g*_ are the respective bulk water and gaseous concentrations, *H* is the dimensionless gas- over-liquid form of the Henry’s law coefficient and *k*_*T*_, *k*_*w*_ and *k*_*a*_ are the total, liquid and air mass transfer coefficients, respectively. We use the Nightingale et al. (2000) parameterization for *k*_*w*_, and for *k*_*a*_ the parameterization recommended by Johnson (2010). Pure water values of *H* from the compilation of Sander (1999) were corrected for salinity using the scheme of Johnson (2010).

### THAMO model

The Tropospheric Halogen Chemistry model (THAMO) is a one-dimensional chemistry transport model with 200 stacked boxes at a vertical resolution of 5 m and a total height of 1 km (see Saiz-Lopez et al. 2008). The lowest level is the ocean surface, where gas phase deposition and upward flux can occur. Deposition to a specified aerosol surface area occurs with uptake coefficients taken from the recommendations of Sander et al. (2006) and Atkinson et al. (2000). The chemical scheme used for this study is from Saiz-Lopez et al. (2008) and is updated according to Mahajan et al. (2010a, b).

The modeled data discussed herein are the THAMO results at a height of 10 m above sea level (asl). The injection of CH_2_I_2_ and CH_2_ICl in the model is constrained by the fluxes measured in the field campaign. Photolysis rate constants for CH_2_I_2_ and CH_2_ICl were calculated by an explicit two-stream radiation scheme, which calculates the solar irradiance as a function of altitude, wavelength and solar zenith angle. For these simulations we assumed clear sky conditions. The modelled loss due to dry deposition of these species to the ocean is about 2 %. Measured O_3_ concentrations and assumed ranges of oceanic NO_2_ mixing ratios (see section 3.3) were used to calculate NO_3_ radical concentrations.

### Laboratory measurements

Kinetics of the NO_3_ + CH_2_I_2_ reaction were derived from a laboratory study conceived primarily to investigate the reaction between stabilised Criegee intermediates (SCIs) and NO_2_. Full details are given in Ouyang et al. (2013); briefly, diiodomethane (CH_2_I_2_) was photolysed at 248 nm, in the presence of NO_2_ and a bath gas of synthetic air. 248 nm CH_2_I_2_ photolysis is known to lead to the formation of the CH_2_OO SCI in the presence of oxygen (Gravestock et al. 2010; Welz et al. 2012), and in turn form NO_3_ as a product of the CH_2_OO + NO_2_ reaction. NO_3_ formation was monitored by broad-band cavity enhanced absorption spectroscopy (BBCEAS), permitting insight into the CH_2_I_2_ + NO_3_ reaction. Experiments were performed across the accessible experimental conditions (in particular, with regard to precursor abundance), including monitoring NO_3_ abundance as a function of the CH_2_I_2_ concentration, whilst keeping all other aspects constant; these data allow us to constrain the rate constant *k*(NO_3_ + CH_2_I_2_).

Experiments were performed in an atmosphere of synthetic air at ambient (1 bar) pressure and 295 ± 5 K temperature, with 450 ppb NO_2_ and with the CH_2_I_2_ mixing ratio varied from 56 to 541 ppb. The reagent mixture was continuously flowed through the reaction cell, and photolysed by the 248 nm output from an Excimer laser, expanded and re-collimated to fill the reaction cell, operating at a total power of approximately 200 mJ pulse^−1^ (monitored by a calibrated power meter located after the reaction cell), and with a pulse repetition frequency of 0.1 Hz (ensuring total gas replenishment between pulses). The resulting NO_3_ formation was measured by BBCEAS, after a ca. 4 s transit time from the photolysis reactor to the absorption instrument. Owing to the presence of substantial concentrations of NO_2_, the majority of the NO_3_ was titrated into N_2_O_5_ during the 4 s transit period; the BBCEAS was therefore operated using a heated inlet and absorption cell, to thermally decompose N_2_O_5_ to NO_3_ + NO_2_. The quantity measured was therefore technically the sum of NO_3_ + N_2_O_5_, as described in the Results.

## Results

### Kinetic data

**Fig. 2:**
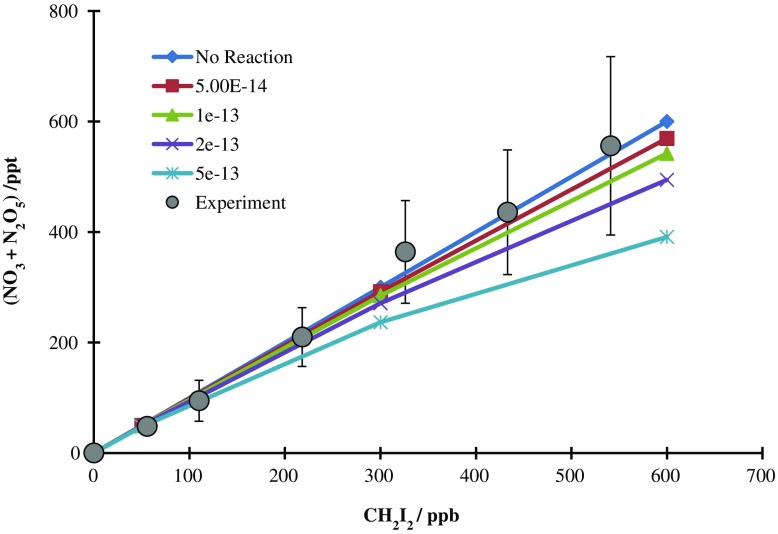
Measured (NO_3_ + N_2_O_5_) mixing ratio (filled grey circles, with ±2 σ error bars, reflecting combined precision and systematic (uncertainties in the NO_3_ absorption spectrum & fitting) components), compared with model predictions of the variation in (NO_3_ + N_2_O_5_) (coloured lines), as a function of CH_2_I_2_, for different values of *k*_1_ (NO_3_ + CH_2_I_2_) in units of cm^3^ molecule^−1^ s^−1^

The measured variation in (NO_3_ + N_2_O_5_) with CH_2_I_2_ abundance is shown in Fig. 2. The observed behaviour was compared with the predictions of a simple model, which reproduced the evolution of NO_3_ between the photolysis pulse and the BBCEAS cavity (i.e*.* through the 4 s transit period), and which incorporated initial NO_3_ production, NO_3_/NO_2_/N_2_O_5_ equilibration, and the reaction of NO_3_ with CH_2_I_2_ to form (unreactive) products. Within the model, the NO_2_ and CH_2_I_2_ concentrations were fixed at their (known) values; standard NO_2_/NO_3_/N_2_O_5_ kinetics were taken from the MCMv3.1 (Bloss et al. 2005), and the initial NO_3_ abundance was calculated based upon the CH_2_I_2_ abundance, laser fluence and SCI yield; a single optimised value of the latter parameter was applied to all simulations. The rate constant for the NO_3_ + CH_2_I_2_ reaction, *k*_1_, was varied, allowing the predicted variation of (NO_3_ + N_2_O_5_) with CH_2_I_2_ concentration to be compared with that observed as shown in Fig. 2. The experimental data clearly show an essentially linear dependence upon CH_2_I_2_, indicating that the NO_3_ + CH_2_I_2_ reaction has little effect under these (laboratory) conditions. The model data show that values of *k*_1_ in excess of 5 × 10^−13^ molec cm^−3^ are not consistent with the observed behaviour, and we derive an upper limit of *k*_1_ ≤ 4 × 10^−13^ molec cm^−3^, at 295 K and 1 bar total pressure (i.e*.* nominal ambient MBL conditions).

### Field data

**Fig. 3:**
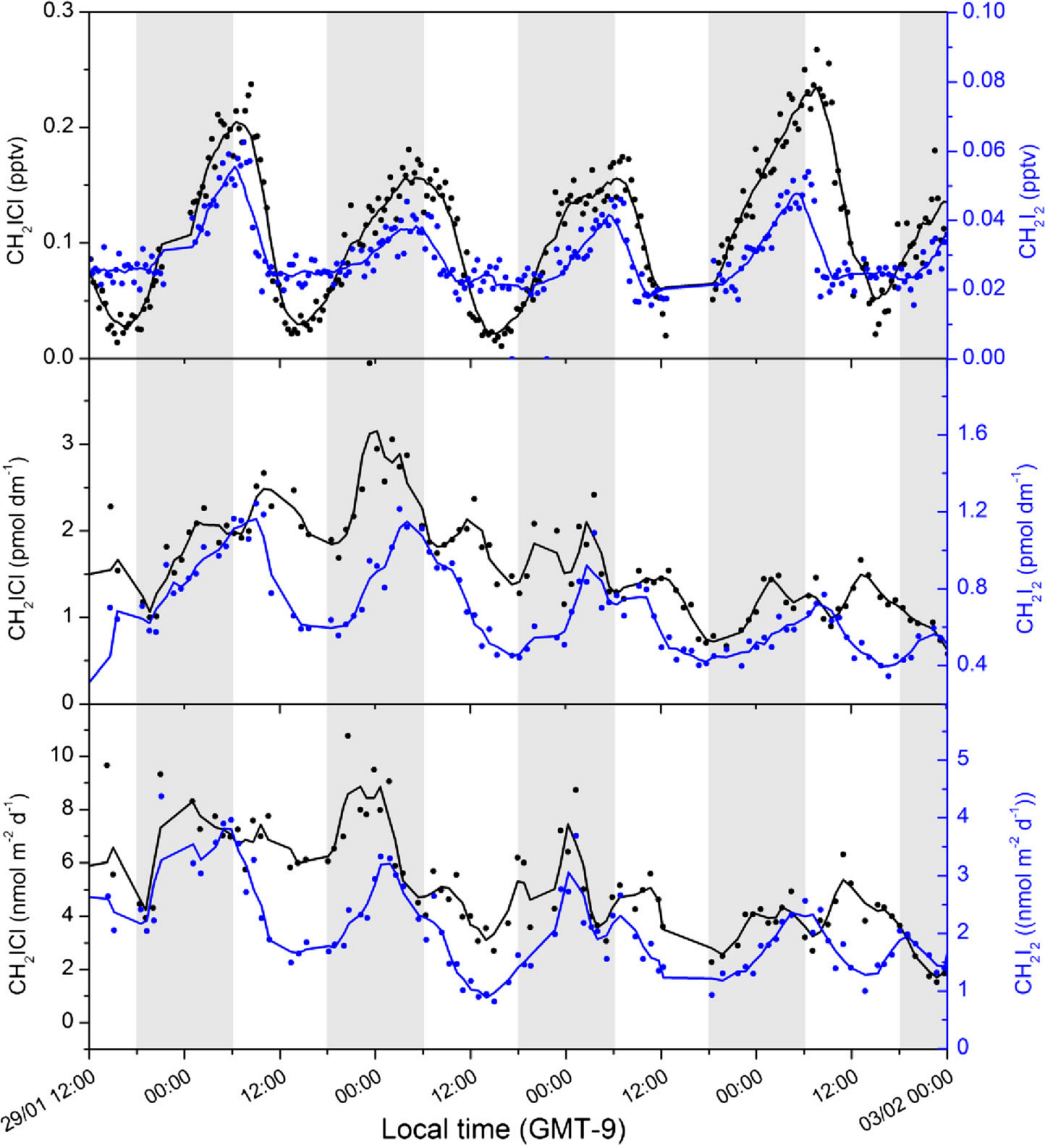
Atmospheric mixing ratios, seawater concentrations, and sea-air fluxes of CH_2_I_2_ and CH_2_ICl measured during the TORERO cruise

We choose as a case study 4 days of measurements during 29th Jan. – 2nd Feb. 2012 whilst transiting the North Equatorial Current in a southeasterly direction (Fig. 1). This period encountered stable surface chlorophyll *a* concentrations (0.1–0.2 μg m^−3^), sea surface temperatures (SSTs) (24.3 ± 0.3 °C), wind speeds (8.5 ± 0.8 ms^−1^) and wind direction (Fig. 1), and showed similar daily patterns of CH_2_I_2_ and CH_2_ICl atmospheric and seawater concentrations (Fig. 3).

Both gases displayed notable diurnal cycles in the atmosphere with mixing ratios steadily increasing during the night and rapidly decreasing at sunrise. Mean night-time/early morning maxima and day-time minima were (max: 0.047 +/− 0.01) pptv and (min: 0.023 +/− 0.004) pptv for CH_2_I_2_ and (max: 0.17 +/− 0.03) pptv and (min: 0.03 +/− 0.01) pptv for CH_2_ICl. Thus, the ratio of night-time maxima to day-time minima was more than a factor of two higher for CH_2_ICl mixing ratios (average of 5.3) than for CH_2_I_2_ (average of 2.0). This is surprising since the atmospheric photolysis lifetime of CH_2_I_2_ is much shorter than that of CH_2_ICl, as discussed in the Introduction. Seawater concentrations also showed diurnal cycles in CH_2_I_2_, as expected due to its fast photochemical decay in seawater (Jones and Carpenter 2005). Fluxes averaged 1.8 +/− 0.8 nmol m^−2^ d^- 1^ for CH_2_I_2_ and 3.7 +/− 0.8 nmol m^−2^ d^- 1^ for CH_2_ICl for the case study period, with night-time fluxes around 2–3 times higher than day-time for CH_2_I_2_ (but only 15 % higher and within the data variability for CH_2_ICl). Thus, in the absence of any night-time loss, nocturnal CH_2_I_2_ mixing ratios should be higher than expected given an assumption of a constant sea-air flux, whereas the reverse is true. Hourly fluxes are used to drive the 1D modeling, so such effects are taken care of in the analysis discussed in the next sections. Together, these results strongly suggest an additional night-time loss of CH_2_I_2_ in the atmosphere.

### 1D modelling

**Fig. 4:**
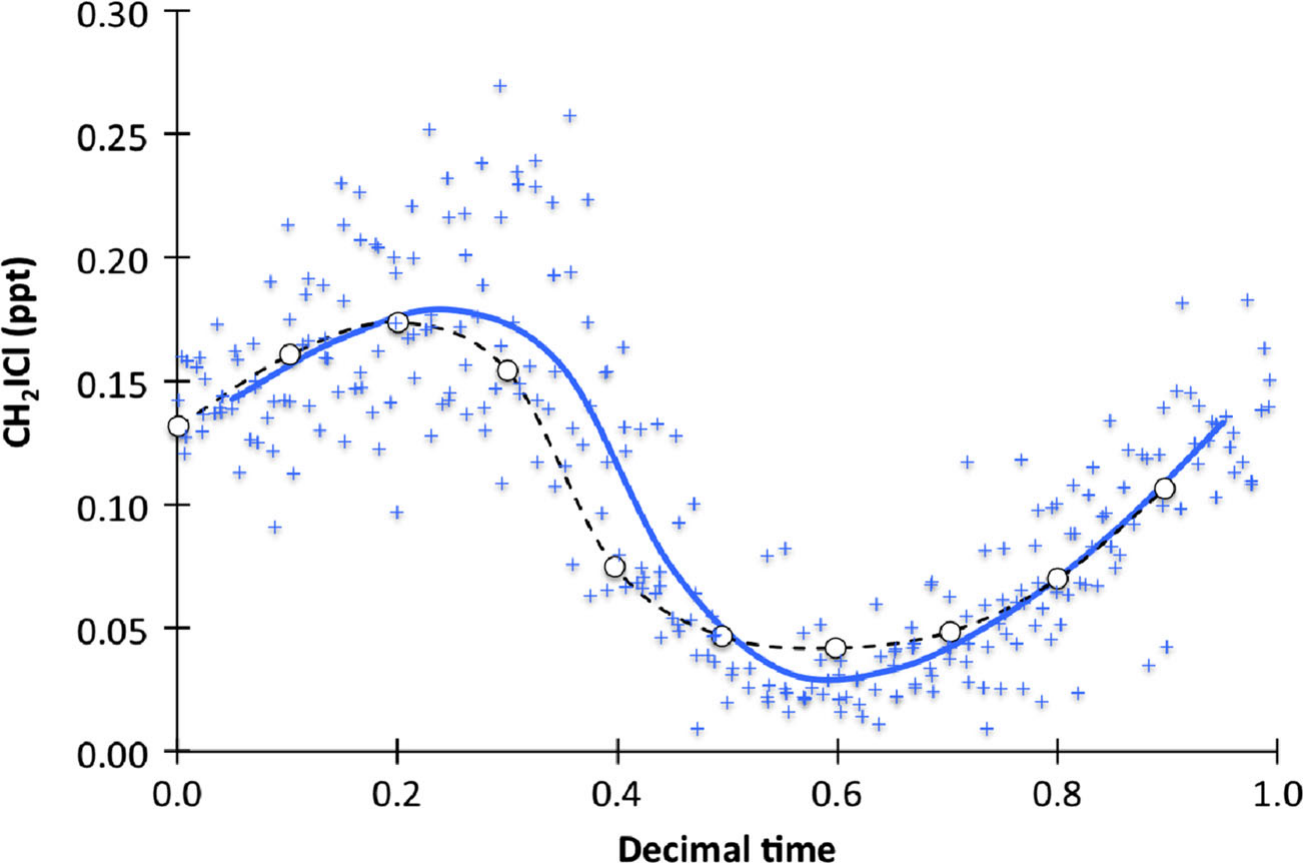
Diurnal average CH_2_ICl mixing ratios during the period 29th Jan. – 2nd February 2012. Circles and black dotted lines show average of THAMO simulations. Blue crosses are hourly measurements, stripped of day of year. Blue line shows an hourly average of the measurements

The THAMO model was used to investigate the effect of reaction (1) on the levels of observed CH_2_I_2_ during the TORERO campaign. Fig. 4 shows that there was no systematic difference between modeled and measured CH_2_ICl mixing ratios, suggesting that the model dynamics and mixing have the required level of skill, and that depositional losses are appropriate. Both the overall CH_2_ICl levels and the diurnal range are simulated well, and no night-time losses of CH_2_ICl are required to reconcile the model output with the observations.

**Fig. 5:**
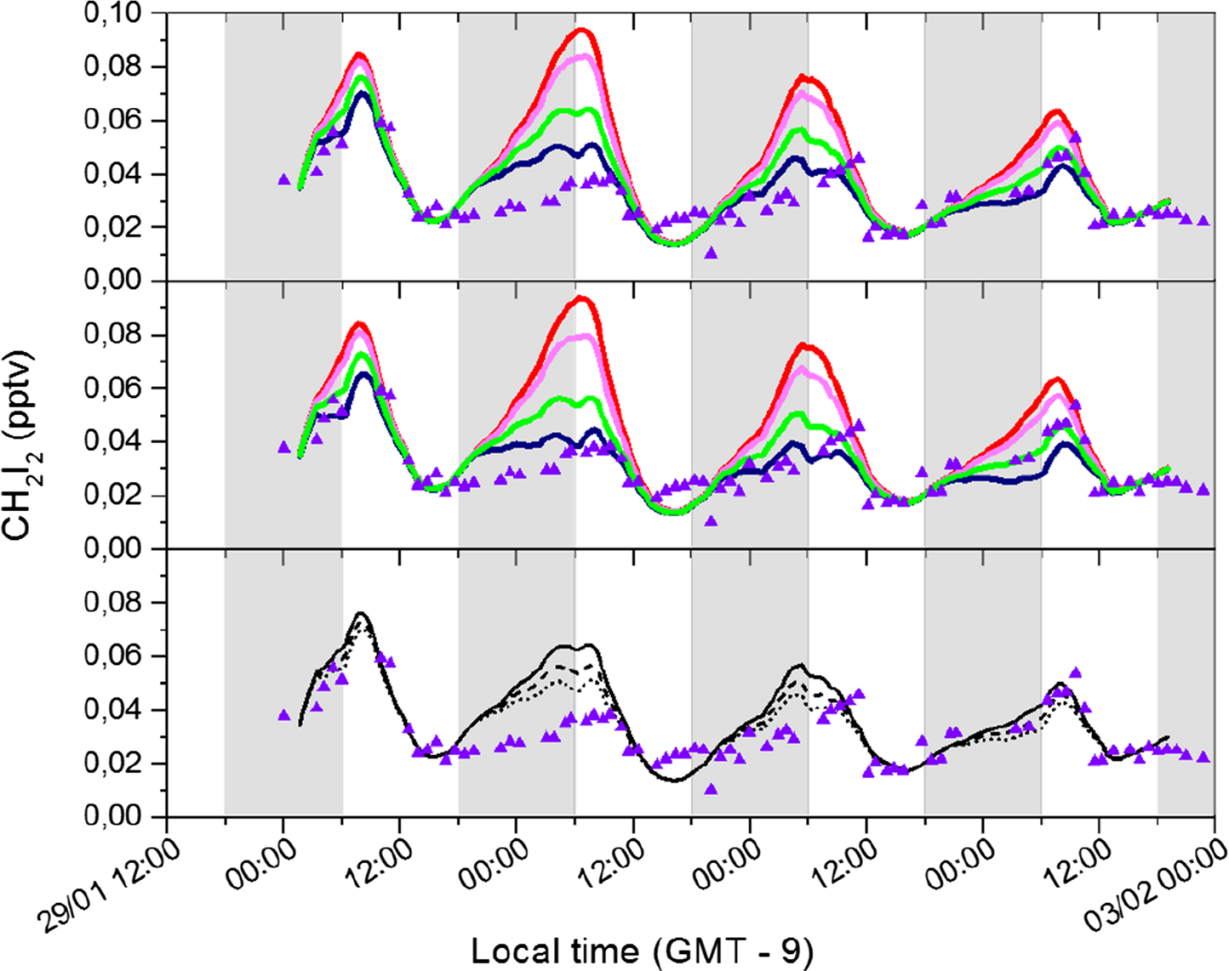
The influence of different *k*_1_ and NO_2_ mixing ratios on modelled CH_2_I_2_, compared with measurements. Lines are model results, purple triangles indicate CH_2_I_2_ measurements. Upper panel: 20 ppt NO_2_ at different *k*_1_ (red - no reaction, magenta – *k*_1_ = 5 × 10^−14^, green - *k*_1_ = 2 × 10^−13^, dark blue - *k*_1_ = 4 × 10^−13^ cm^3^ molecule^−1^ s^−1^); Middle panel: 30 ppt NO_2_ at different *k*_1_ (lines as before); Lower panel: varying NO_2_ concentrations at *k*_1_ = 2 × 10^−13^ cm^3^ molecule^−1^ s^−1^ (solid line - NO_2_ = 20 ppt, dashed line - NO_2_ = 30 ppt; dotted line - NO_2_ = 40 ppt)

Figure 5 shows however that for CH_2_I_2_, the base model (no NO_3_ reaction) overestimates night-time mixing ratios. On average over this period the model overestimation is a factor of 1.62. Figure 5 also shows the influence of including reaction (1) at different rate constants and NO_2_ (hence NO_3_ radical) concentrations.

Three out of the four days of data are simulated well with *k*_1_ of 2 × 10^−13^ cm^3^ molecule^−1^ s^−1^ and 30–40 pptv NO_2_ (corresponding to 14–18 ppt peak NO_3_) or 4 × 10^−13^ cm^3^ molecule^−1^ s^−1^ and 20 pptv NO_2_ (corresponding to 9 ppt peak NO_3_). On the 31st January the model overestimates average CH_2_I_2_ night-time levels under all the scenarios considered here, although the maximum night-time concentration is simulated well under the highest loss scenario. Without additional measurements we cannot be conclusive on the reasons for this, although higher NO_3_ radical concentrations during this period are a possible explanation. Aircraft measurements made over the eastern Pacific Ocean as part of the NASA GTE/CITE 2 program indicate typical NO_2_ values of 18 +/− 15 ppt for maritime tropical air (Carroll et al. 1990) and ~50 ppt has been estimated as an upper limit for the remote ocean boundary layer from MAX-DOAS and satellite (SCIAMACHY and GOME-2) data (Peters et al. 2012).

## Summary and conclusions

Our field data of atmospheric CH_2_I_2_ along with oceanic fluxes calculated from measured seawater concentrations are strongly indicative of a missing night-time sink of CH_2_I_2_, potentially through reaction with NO_3_ radicals. In the absence of NO_3_ measurements during our field study, it is not possible to infer the CH_2_I_2_ + NO_3_ rate constant with certainty, although a range of 2–4 × 10^−13^ cm^3^ molecule^−1^ s^−1^ offers generally good reconciliation between model and measurements. This range is consistent with a laboratory-derived upper limit, presented herein, and previous direct measurements made at low pressure. Given the importance of this reaction for understanding the atmospheric behavior of CH_2_I_2_, we recommend further laboratory measurements of the rate constant and the products of the reaction. Nakano et al. (2006) suggest that the possible products may be the compounds produced from H- transfer and I-transfer reactions:

1a $$ {\mathrm{NO}}_3+{CH}_2{\mathrm{I}}_2\to {HNO}_3+{\mathrm{CHI}}_2 $$

1b $$ {\mathrm{NO}}_3+{CH}_2{\mathrm{I}}_2\to {\mathrm{I}\mathrm{ONO}}_2+{CH}_2\mathrm{I} $$

The available thermodynamic data suggest that channel 1a is favourable but 1b is endothermic (Atkinson et al. 2004; Marshall et al. 2005; Sander et al. 2006), indicating that the reaction NO_3_ + CH_2_I_2_ represents a loss of NO_x_ to HNO_3_. Further reactions of CHI_2_ and CH_2_I are uncertain, although in both cases I atoms are eventually produced which will form IO in the presence of O_3_. Oxidation of CH_2_I (by O_2_) leads to the Criegee intermediate CH_2_OO along with other products (Gravestock et al. 2010; Stone et al., 2013 and references therein). In locations with high CH_2_I_2_ emissions and sufficient NO_3_, such as polluted coastlines, the reaction may result in production of nighttime IO, or at least in modification of the diurnal profile of IO.
